# Supplementary material for: A Spiking Neuron and Population Model Based on the Growth Transform Dynamical System
Source: Front Neurosci. 2020 May 12;14:425. doi: 10.3389/fnins.2020.00425 (PMC7235464; doi:10.3389/fnins.2020.00425)
Supplement: Supplementary file 1 [file Data_Sheet_1.pdf]

Appendix

APPENDIX A

We can rewrite (1) for a sequence $\left(v_{i,n-N+1}, v_{i,n-N+2}, ..., v_{i,n}\right)$ of size N to obtain

$$
\begin{aligned}
v_{i,n-N+2} &= v_{i,n-N+1} + (\gamma-1)v_{i,n-N+1} + \sum_{j=1}^{M} W_{ij}\Psi(v_{j,n-N+1}) + y_{i,n-N+1} \\
v_{i,n-N+3} &= v_{i,n-N+2} + (\gamma-1)v_{i,n-N+2} + \sum_{j=1}^{M} W_{ij}\Psi(v_{j,n-N+2}) + y_{i,n-N+2} \\
&. \\
&. \\
v_{i,n+1} &= v_{i,n} + (\gamma-1)v_{i,n} + \sum_{j=1}^{M} W_{ij}\Psi(v_{j,n}) + y_{i,n}
\end{aligned}
$$

Summing over the time-steps and dividing by the total number of time-steps, we get

$$
(1-\gamma)\mathbb{E}_N\Big(v_i[n]\Big) + \frac{1}{N}\Big(v_{i,n+1} - v_{i,n-N+1}\Big) = \sum_{j=1}^{M} W_{ij}\mathbb{E}_N\Big(\Psi_j[n]\Big) + \mathbb{E}_N\Big(y_i[n]\Big), \tag{A.1}
$$

where $\mathbb{E}_N\Big(\Psi_j[n]\Big) = \frac{1}{N}\sum\limits_{p=n-N+1}^{n}\Psi(v_{j,p})$. Since the neural responses are assumed to be bounded at all times, as $N \to \infty$, the second term in (A.1) approaches zero, so that we can rewrite (A.1) as

$$
(1-\gamma)\bar{v}_i[n] = \sum_{j=1}^{M} W_{ij}\bar{\Psi}_j[n] + \bar{y}_i[n]. \tag{A.2}
$$

APPENDIX B

Proof of Proposition I: We can decompose the scalar variable $v_{i,n}$ as $v_{i,n} = v_{i,n}^{+} - v_{i,n}^{-}$, where $v_{i,n}^{+}, v_{i,n}^{-} \geq 0$. The following additional constraint

$$
v_{i,n}^{+} + v_{i,n}^{-} = v_c, \quad i = 1, ..., M, \tag{B.1}
$$

imposed on the variables $v_{i,n}^{+}$ and $v_{i,n}^{-}$ would then always ensure that (10) is satisfied. Then we can write

$$
\underset{|v_i|\leq v_c}{\arg\min}\ \mathcal{H}\big(\{v_i\}\big) \equiv \underset{v_i^{+}+v_i^{-}=v_c;\ v_i^{+},v_i^{-}\geq 0}{\arg\min}\ \mathcal{H}\big(\{v_i^{+} - v_i^{-}\}\big), \tag{B.2}
$$

We can solve the equivalent optimization problem under the linear constraint specified by (B.1) and the non-negativity constraints, using an iterative multiplicative update called Growth Transforms - a fixed

point algorithm for optimizing a Lipschitz continuous objective function under similar equality constraints (Gopalakrishnan et al., 1989; Chatterjee and Chakrabartty, 2018).

Baum-Eagon Growth Transformations: Baum-Eagon Growth Transforms (Chatterjee and Chakrabartty, 2018) are a class of fixed-point algorithms for iteratively optimizing a Lipschitz continuous objective function $\mathcal{H}(\{v_{ik}\})$ that is constrained over a domain $\mathcal{D}$ defined by

$$\mathcal{D} = \Big\{v_{ik} : v_{ik} \geq 0 \ \text{ and } \ \sum_{k=1}^{q_i} v_{ik} = v_c\Big\}, \tag{B.3}$$

where $q_1, q_2, ..., q_M$ is a set of non-negative integers and M is a positive integer which denotes the number of linear constraints in $\mathcal{D}$. For a Lipschitz continuous cost function $\mathcal{H}(\{v_{ik}\})$ where $v_{ik} \in \mathcal{D}, \ \ i = 1, ..., M$, there exists a growth transformation $\sigma(.)$ such that

$$\mathcal{H}\big(\{\sigma(v_{ik})\}\big) \leq \mathcal{H}\big(\{v_{ik}\}\big) \ \ \forall v_{ik} \in \mathcal{D}. \tag{B.4}$$

This transformation takes the following form

$$\sigma(v_{ik,n}) = v_{ik,n+1} \leftarrow v_c \frac{G_{ik}(\boldsymbol{v}_n, \lambda)}{G_i(\boldsymbol{v}_n, \lambda)}, \ \ i = 1, ..., M, \tag{B.5}$$

where

$$G_{ik}(\boldsymbol{v}_n, \lambda) \ = \ v_{ik,n}\big(- \frac{\partial \mathcal{H}}{\partial v_{ik,n}} + \lambda\big), \ \text{ and} \tag{B.6}$$

$$G_i(\boldsymbol{v}_n, \lambda) \ = \ \sum_{k=1}^{q_i} G_{ik}(\boldsymbol{v}_n, \lambda). \tag{B.7}$$

An admissible value for the constant λ is such that for any $v_{ik,n} \in \mathcal{D}$, $G_{ik}(\boldsymbol{v}_n, \lambda) \geq 0$ and $G_i(\boldsymbol{v}_n, \lambda) > 0$ (Gopalakrishnan et al., 1989).

Growth Transform neuron model updates. Assuming that the partial derivatives for the network energy functional in (8) are bounded, the optimization problem for the proposed model is equivalent to the one in (B.2), being constrained on a domain equivalent to $\mathcal{D}$, where $q_1 = q_2 = ... = q_M = 2$, and $v_i^+ + v_i^- = v_c, \ \ i = 1, ..., M$ are the M linear constraints. Considering $v_i^+ = v_{i1}$ and $v_i^- = v_{i2}$, we can rewrite the update equations in (B.5) in terms of the new optimization variables corresponding to (B.2) to obtain the following discrete-time updates

$$v_{i,n+1}^+ \ = \ v_c \frac{v_{i,n}^+}{\mu_{i,n}}\big(- \frac{\partial \mathcal{H}}{\partial v_{i,n}^+} + \lambda\big) \tag{B.8}$$

$$v_{i,n+1}^- \ = \ v_c \frac{v_{i,n}^-}{\mu_{i,n}}\big(- \frac{\partial \mathcal{H}}{\partial v_{i,n}^-} + \lambda\big), \tag{B.9}$$

where

$$\mu_{i,n} = v_{i,n}^+\big(- \frac{\partial \mathcal{H}}{\partial v_{i,n}^+} + \lambda\big) + v_{i,n}^-\big(- \frac{\partial \mathcal{H}}{\partial v_{i,n}^-} + \lambda\big) \tag{B.10}$$

is a normalization factor that ensures $v^+_{i,n+1} + v^-_{i,n+1} = v_c$. Here, λ (which has the unit of current) is admissible iff $\lambda > |\frac{\partial \mathcal{H}}{\partial v_{i,n}}| \ \forall i, n$, which will ensure $G^+_i(\boldsymbol{v}_n, \lambda) > 0\ W$ and $G^-_i(\boldsymbol{v}_n, \lambda) > 0\ W\ \ \forall i$. From (B.8) and (B.9), using $v_{i,n} = v^+_{i,n} - v^-_{i,n}$ and (B.1), we can easily show

$$v_{i,n+1} = v_c \frac{-\frac{\partial \mathcal{H}}{\partial v_{i,n}} v_c + \lambda v_{i,n}}{-\frac{\partial \mathcal{H}}{\partial v_{i,n}} v_{i,n} + \lambda v_c}\ , \tag{B.11}$$

where we have used the relation $\frac{\partial \mathcal{H}}{\partial v_i} = \frac{\partial \mathcal{H}}{\partial v^+_i} = -\frac{\partial \mathcal{H}}{\partial v^-_i}$. This dynamical system model, derived from the Growth Transform updates outlined in (B.8) and (B.9), ensures that (11) holds, with equality being satisfied iff $v_{i,n}$ is a critical point of $\mathcal{H}$.

Rearranging the terms in (B.11), we get

$$(v_c^2 - v_{i,n} v_{i,n+1}) \frac{\partial \mathcal{H}}{\partial v_{i,n}} + \lambda v_c (v_{i,n+1} - v_{i,n}) = 0. \tag{B.12}$$

Rewriting B.12 for a sequence of time-indices $p = n - N + 1, n - N + 2, ..., n$, of size N, we get

$$\begin{aligned}
(v_c^2 - v_{i,n-N+1} v_{i,n-N+2}) \frac{\partial \mathcal{H}}{\partial v_{i,n-N+1}} + \lambda v_c (v_{i,n-N+2} - v_{i,n-N+1}) &= 0 \\
(v_c^2 - v_{i,n-N+2} v_{i,n-N+3}) \frac{\partial \mathcal{H}}{\partial v_{i,n-N+2}} + \lambda v_c (v_{i,n-N+3} - v_{i,n-N+2}) &= 0 \\
&. \\
&. \\
(v_c^2 - v_{i,n} v_{i,n+1}) \frac{\partial \mathcal{H}}{\partial v_{i,n}} + \lambda v_c (v_{i,n+1} - v_{i,n}) &= 0
\end{aligned}$$

Summing over the time-steps and dividing by the total number of time-steps, we get

$$\mathbb{E}_N \Big(z_i[n] \Big) + \frac{1}{N} \lambda v_c (v_{i,n+1} - v_{i,n-N+1}) = 0 \tag{B.13}$$

where $z_{i,n} = (v_c^2 - v_{i,n} v_{i,n+1}) \frac{\partial \mathcal{H}}{\partial v_{i,n}}$. As $N \rightarrow \infty$, since $v_{i,n}$ are bounded $\forall i, n$, we have for the n-th time-window

$$\lim_{N \rightarrow \infty} \Bigg(\mathbb{E}_N \Big(z_i[n] \Big) \Bigg) \rightarrow 0. \tag{B.14}$$

APPENDIX C

Asymptotic encoding for non-saturating GT neurons:, For neurons with responses $v_{i,n} > -v_c\ \ \forall n$ (which includes spiking neurons as well as non-spiking neurons that do not cross the threshold), we define $\alpha_{i,n} = (v_c^2 - v_{i,n} v_{i,n+1})$. Since $|v_{i,n}| < v_c\ \forall n$, $\alpha_{i,n} > 0\ \forall n$. We can sum the criterion (B.12) for time-steps $p = n - N + 1, n - N + 2, ..., n$, to write

$$\sum_{p=n-N+1}^{n} \alpha_{i,p} g_{i,p} = \lambda v_c (v_{i,n-N+1} - v_{i,n+1}), \tag{C.1}$$

where $g_{i,p} = \frac{\partial \mathcal{H}}{\partial v_{i,p}}$. For $p = n - N + 1, n - N + 2, ..., n$, we can decompose the instantaneous gradient term $g_{i,p}$ as follows

$$g_{i,p} = \mathbb{E}_N\big(g_i[n]\big) + \Delta g_{i,p}, \tag{C.2}$$

where $\Delta g_{i,p}$ is a zero-mean sequence such that $\mathbb{E}_N\big(\Delta g_i[n]\big) = 0$. Then combining (C.1) and (C.2),

$$\begin{aligned}
\sum_{p=n-N+1}^{n} \alpha_{i,p}\mathbb{E}_N\big(g_i[n]\big) + \sum_{p=n-N+1}^{n} \alpha_{i,p}\Delta g_{i,p} &= \lambda v_c(v_{i,n-N+1} - v_{i,n+1}) \\
\mathbb{E}_N\big(g_i[n]\big) \sum_{p=n-N+1}^{n} \alpha_{i,p} &= -\sum_{p=n-N+1}^{n} \alpha_{i,p}\Delta g_{i,p} + \lambda v_c(v_{i,n-N+1} - v_{i,n+1}) \\
\mathbb{E}_N\big(g_i[n]\big) &= -\frac{\sum\limits_{p=n-N+1}^{n} \alpha_{i,p}\Delta g_{i,p}}{\sum\limits_{p=n-N+1}^{n} \alpha_{i,p}} + \frac{\lambda v_c(v_{i,n-N+1} - v_{i,n})}{\sum\limits_{p=n-N+1}^{n} \alpha_{i,p}}
\end{aligned}$$

Since $\alpha_{i,n} > 0 \;\; \forall n$, $\sum\limits_{p=n-N+1}^{n} \alpha_{i,p} \rightarrow \infty$ as $N \rightarrow \infty$. Also, $|v_{i,n}| < v_c \;\; \forall n$ leads to

$$\lim_{N\rightarrow\infty}\Bigg(\mathbb{E}_N\big(g_i[n]\big)\Bigg) = \lim_{N\rightarrow\infty}\Bigg(-\frac{\sum\limits_{p=n-N+1}^{n} \alpha_{i,p}\Delta g_{i,p}}{\sum\limits_{p=n-N+1}^{n} \alpha_{i,p}}\Bigg) = 0. \tag{C.3}$$

The last result uses the non-pathological case that due to the bounded property of $\Delta g_{i,p}$, the sequence $\alpha_{i,p}\Delta g_{i,p}$ does not grow as fast as the denominator sequence $\alpha_{i,p}$.

APPENDIX D

Continuous-time Growth Transform dynamical system: In order to derive the complete dynamical system model for the Growth Transform neuron, we apply a useful property of Growth Transforms. The Growth Transform mapping $\sigma(.)$ homotopically minimizes the value of the cost function $\mathcal{H}$ (Baum and Sell, 1968) as shown below

$$\mathcal{H}\Big(\{(1 - f_{i,n})v_{i,n}^{\zeta} + f_{i,n}\sigma(v_{i,n}^{\zeta})\}\Big) \leq \mathcal{H}\Big(\{v_{i,n}^{\zeta}\}\Big), \;\; \zeta = +, -; \tag{D.1}$$

where $0 < f_{i,n} \leq 1$. This leads to the updated discrete-time equations for the new optimization variables for minimizing $\mathcal{H}(.)$

$$v_{i,n+1}^{+} = (1 - f_{i,n})v_{i,n}^{+} + f_{i,n}v_c\frac{v_{i,n}^{+}}{\mu_n}(-\frac{\partial \mathcal{H}}{\partial v_{i,n}^{+}} + \lambda) \tag{D.2}$$

$$v_{i,n+1}^{-} = (1 - f_{i,n})v_{i,n}^{-} + f_{i,n}v_c\frac{v_{i,n}^{-}}{\mu_n}(-\frac{\partial \mathcal{H}}{\partial v_{i,n}^{-}} + \lambda). \tag{D.3}$$

From (B.11), (D.2) and (D.3), we have

$$v_{i,n+1} = (1 - f_{i,n})v_{i,n} + f_{i,n}v_c \frac{-\frac{\partial \mathcal{H}}{\partial v_{i,n}} v_c + \lambda v_{i,n}}{-\frac{\partial \mathcal{H}}{\partial v_{i,n}} v_{i,n} + \lambda v_c}. \tag{D.4}$$

We define $\tau_{i,n} = \Delta t(1/f_{i,n} - 1)$, where Δt is the time-increment in seconds between two time-steps as defined previously in (1). Then since $0 < f_{i,n} \leq 1$, we have $\tau_{i,n} \in [0, \infty)$ s. $\tau_{i,n}$ can be considered to be the time-constant for the i-th neuron at the n-th time-step, and the discrete-time dynamical systems model in (D.4) can be written as

$$(\tau_{i,n} + \Delta t)\left(\frac{v_{i,n+1} - v_{i,n}}{\Delta t}\right) + v_{i,n} = v_c \frac{-\frac{\partial \mathcal{H}}{\partial v_{i,n}} v_c + \lambda v_{i,n}}{-\frac{\partial \mathcal{H}}{\partial v_{i,n}} v_{i,n} + \lambda v_c}. \tag{D.5}$$

Since the n-th time-step corresponds to time $t = n\Delta t$, $v_{i,n} \equiv v_i(n\Delta t)$ and (D.5) can be rewritten as

$$(\tau_i(t) + \Delta t)\left(\frac{v_i(t + \Delta t) - v_i(t)}{\Delta t}\right) + v_i(t) = v_c \frac{-\frac{\partial \mathcal{H}}{\partial v_i(t)} v_c + \lambda v_i(t)}{-\frac{\partial \mathcal{H}}{\partial v_i(t)} v_i(t) + \lambda v_c} \tag{D.6}$$

In the limiting case when $\Delta t \to 0$ s, this reduces to the following continuous-time dynamical system model (Chatterjee and Chakrabartty, 2018)

$$\tau_i(t)\frac{dv_i(t)}{dt} + v_i(t) = v_c \frac{-\frac{\partial \mathcal{H}}{\partial v_i(t)} v_c + \lambda v_i(t)}{-\frac{\partial \mathcal{H}}{\partial v_i(t)} v_i(t) + \lambda v_c}. \tag{D.7}$$

REFERENCES

Baum, L. E. and Sell, G. (1968). Growth transformations for functions on manifolds. *Pacific Journal of Mathematics* 27, 211–227

Chatterjee, O. and Chakrabartty, S. (2018). Decentralized global optimization based on a growth transform dynamical system model. *IEEE Transactions on Neural Networks and Learning Systems*

Gopalakrishnan, P., Kanevsky, D., Nadas, A., and Nahamoo, D. (1989). A generalization of the baum algorithm to rational objective functions. In *International Conference on Acoustics, Speech, and Signal Processing, 1989.* (IEEE), 631–634
